# Account of the Efficacy of Copious Blood-Letting in a Wound of the Lungs, &c.

**Published:** 1807-10

**Authors:** Hugh G. Shaw


### *Account of the Efficacy of copious Blood-letting in a Wound of the Lungs, &c. By* Dr. Hugh G. Shaw; *in a Letter to* Dr. Rush.

Dear Sir,

I EARLY had it in contemplation to communicate to you the following remarkable case of a wound in the lungs, which came under Dr. Martin's and my observation, the last summer. Your late request to the Doctor, has confirmed me in my intention; I therefore proceed to lay it before you, relying on your usual candour to point out its imperfections.

Henry Oelman, an active man, about forty years of age, a native of Germany, on the evening of the 18th of June last, in attempting to jump from a hay-loft window into a waggon, fell upon one of the ledge-pins, which penetrated between the second and third false ribs of the right side, in an oblique direction upwards. The seat of the accident being at Mr. Abraham Rittenhouse's, two miles from Germantown, an hour had nearly elapsed before we saw him. On inquiring into particulars, his companions of the meadow informed us, "that he had turned half round on the pin, in order to disengage himself, before they could relieve him; and that a stout man, with much difficulty, raised him off, supported in his arms." On examining the wound and the instrument, we found it had passed eight-and-a-half inches, (that extremity being three and one-eighth inches in circumference) and, in its progress, wounded the anterior portion of the right lobe of the lungs.

This we were satisfied in, from plainly distinguishing with the finger, the lacerated parts, when we placed him in different positions.

The intercostal artery, although doubtless abraded, was not ruptured; the hemorrhage, however, was considerable. On withdrawing the finger, a great quantity of air rushed from the wound. Having made use of the common method thod of expelling the remainder of what entered there, we dressed it with a pledget of simple ointment. At this time, the hemorrhage externally, had stopped. He complained of violent oppression; his pulse tense and depressed, his breathing very difficult, though he spoke pretty distinctly. As quickly as circumstances would admit, we took thirty-two ounces of blood from him, after which he said he felt greatly relieved. In order to empty the intestines, he was afterwards ordered a smart purge. The next morning at five o'clock we saw him. His purge had operated, and he slept tolerably well the remainder of the night; but then complained of a severe pain and oppression across the inferior part of the thorax, as far as the scrobiculus cordis. The blood drawn the evening before was not remarkably sizy. I immediately took away eighteen ounces more; his pulse, which before was uncommonly tense, now became softer and fuller, and he said he was much easier. I discovered that one of the family, in order to gratify his accustomed fondness for strong liquors, and willing, as he said, to comfort him in his last moments, had given him a glass or two of spirits. We took the alarm, and determining to adhere closely to the antiphlogistic plan, strictly enjoined the necessity of avoiding any thing the least spirituous, and ordered him to live on weak gruel, chicken water, &c. which I believe, except in one instance, was punctually complied with. I confess I was remiss in not enforcing this earlier. We immediately put him on the use of nitre and the vegetable acids. We were fearful of joining tart. emet. to the nitre, lest the quantity necessary to support a gentle diaphoresis might excite vomiting. We ordered, as a laxative, occasional doses of the ol. ricini, which he said he could take better than any other medicine. At twelve o'clock, I found his pulse hard, accompanied with no small degree of dyspnœa. The seat of the most considerable pain was posteriorly along the lower false ribs. This I attributed to an accumulation of extravasated blood. I took away ten ounces, dressed the wound and found its appearance favourable. In the evening, I observed that the blood taken at noon was much more sizy than either of the preceding. From this circumstance, and the still existing painful and oppressive symptoms, I was readily induced to repeat the bleeding to the amount of ten ounces. This bleeding, he said, sensibly relieved him, more particularly his breathing. Saturday morning, 20th, I found him much better; the wound discharging a laudable pus; his pulse pretty regular, breathing tolerable able easy; almost clear of pain; his bowels quite free. I therefore did not bleed him, but I had afterwards reason to repent that I had not; for at twelve o'clock, a messenger came up and told me "he was dying!" I went down immediately, and found him in violent pain, delirious, with a quick tense pulse, and great difficulty of breathing. On applying the ligature with a view to take more blood, I was surprised to find that it would not flow as usual, from the first orifice; I immediately enlarged the orifice, to twice the size, when to my astonishment, the blood passed off in a thick and grumous state, with considerable large clots. I was convinced from this appearance, that a re-absorption had taken place. After losing twelve ounces he declared himself "another man." Dr. Martin in the evening found his pulse at 85, and full; his appetite remarkably good, as from the beginning. He took away eight ounces of blood, still dark and grumous; and ordered him some fruit jellies. Sunday, 21st, his pulse unusually full and quick. On inquiry, I found that he had eaten meat, contrary to our strictest orders. I took away ten ounces of blood, which ran freely from the first orifice, and appeared much thinner than any of the preceding. He continued pretty easy all that day and the next, requiring only two small bleedings (sixteen ounces). Tuesday, 23d, rested very well the night before; his pulse soft and regular; appetite good; so well as to sit in a chair and have the wound dressed, which was now nearly closed. Wednesday, Continues free from pain, except a little in the right shoulder. Thursday, 25th, One of his friends came to me in a great hurry, and told me he was dangerously ill. On my arrival, I found his situation truly alarming: a strong hard pulse, skin and tongue dry, violent pain in the head and breast, dyspnœa, delirium. On inquiring into the cause of this dreadful change, the family informed me, "that he had imprudently dressed himself, the day before, and sat by an open window, while it rained; that he frequently put his head out and got wet." I once more had recourse to the lancet, and took away twenty-eight ounces of the purple fluid; on which he exclaimed, "that he felt himself in a new world." In the evening he told me the mercurial pills I ordered had operated very well, but that he still felt a good deal of pain and oppression; his pulse full, and veins turgid. I bled him twenty-four ounces, which instantly relieved his pain and oppressed respiration.

The

The two succeeding days passed without any symptom of danger or alarm. Sunday, 27th, Dr. Martin visited him; found his pulse full, and about 80; had slept tolerably; the wound almost healed; took away ten ounces. At half past three I found him cheerful; his pulse 75; had two evacuations from having taken some castor oil in the morning. He said the only pain was two inches transversely from the wound. The blood taken in the morning contained a considerable proportion of crassamentum, with but little serum. Half past nine, continues better, though there is a considerable degree of action in his pulse. He sat in a chair and was bled (ad deliquium) eight ounces; appearance black and grumous. His pulse rose considerably after the bleeding. Some pain still continued in his right shoulder. Monday, 28th, Complains of great weakness; pulse is quick, but not full. I took six ounces of blood away, after which he assured me he was entirely clear of pain, except, as he expressed it, " a little between the meat and the ribs;" the wound cicatrizing. The next day I found him sitting up, and in high spirits. He said he felt well enough to ride home, which was half a mile, and anxiously wished to obtain permission; this I peremptorily refused. I continued to visit him for three days. On the fourth I was much surprized, in passing his own house, to see him at the window. On my reproving him, he apologized, by answering, that he became impatient to get home to his family, and had rode up that morning without the least inconvenience. In twenty days from the accident, he made a pair of shoes. We have been under the necessity of bleeding him several times since, at seven or eight weeks interval.

Many cases have since offered, which indispensably required venesection; particularly that of Miss Norton, at the Rising Sun, who, in six days, lost one hundred ounces of blood: both tables of the frontal bone having been dreadfully fractured by the oversetting of a chair.

*Germantown, 24th February,* 1796.

